# Functional Limitations Over Time: The Effects of Socioeconomic Status in Mexico

**DOI:** 10.1093/geroni/igaf122.1162

**Published:** 2025-12-31

**Authors:** Chengming Han, Joey Saavedra, Brian Downer

**Affiliations:** UTMB Health, The University of Texas Medical Branch, Galveston, Texas, United States; UTMB Health, The University of Texas Medical Branch, Galveston, Texas, United States; UTMB, Galveston, Texas, United States

## Abstract

**Objective:**

To examine the longitudinal effects of socioeconomic status on functional limitations in Mexico by comparing two birth cohorts aged 50-58 years.

**Methods:**

Data were drawn from the Mexican Health and Aging Study (MHAS). Functional limitations were measured by the number of mobility difficulties. Cohort 1 (C1, N = 4,450) was interviewed in 2003 and followed up in 2012, while Cohort 2 (C2, N = 4,167) was interviewed in 2012 and followed up in 2021. Multilevel logistic models were used to assess the association between socioeconomic status and functional limitations over time.

**Results:**

Cluster analysis categorized participants into four socioeconomic groups in each cohort: (1) low education and low income (lower class), (2) low education and medium income, (3) high education and medium income, and (4) high education and high income. Regression results showed that the lower class in the 2012 cohort had fewer functional limitations than their counterparts in the 2003 cohort. The other three groups exhibited similar patterns across cohorts. Higher education (high school or college) was associated with fewer functional limitations. Women engaged in housework had greater functional limitations. Higher income was protective against functional limitations, but only for women.

**Conclusion:**

Over recent decades, lower-class individuals in the 2012 cohort experienced fewer functional limitations compared to those in the 2003 cohort. Educational attainment consistently showed protective effects, while income and occupational influences on functional limitations varied by gender.

